# A Machine Learning Approach for Highlighting microRNAs as Biomarkers Linked to Amyotrophic Lateral Sclerosis Diagnosis and Progression

**DOI:** 10.3390/biom14010047

**Published:** 2023-12-29

**Authors:** Graziantonio Lauria, Rosita Curcio, Paola Tucci

**Affiliations:** Department of Pharmacy, Health and Nutritional Sciences, University of Calabria, 87036 Rende, Italy; graziantonio.lauria@unical.it

**Keywords:** ALS, miRNAs, degenerative diseases, clinical markers, prognosis, text mining, digitalization

## Abstract

Amyotrophic Lateral Sclerosis (ALS) is a fatal neurodegenerative disease characterized by the progressive loss of motor neurons in the brain and spinal cord. The early diagnosis of ALS can be challenging, as it usually depends on clinical examination and the exclusion of other possible causes. In this regard, the analysis of miRNA expression profiles in biofluids makes miRNAs promising non-invasive clinical biomarkers. Due to the increasing amount of scientific literature that often provides controversial results, this work aims to deepen the understanding of the current state of the art on this topic using a machine-learning-based approach. A systematic literature search was conducted to analyze a set of 308 scientific articles using the MySLR digital platform and the Latent Dirichlet Allocation (LDA) algorithm. Two relevant topics were identified, and the articles clustered in each of them were analyzed and discussed in terms of biomolecular mechanisms, as well as in translational and clinical settings. Several miRNAs detected in the tissues and biofluids of ALS patients, including blood and cerebrospinal fluid (CSF), have been linked to ALS diagnosis and progression. Some of them may represent promising non-invasive clinical biomarkers. In this context, future scientific priorities and goals have been proposed.

## 1. Introduction

Amyotrophic Lateral Sclerosis (ALS) is a progressive neurodegenerative disorder that affects nerve cells in the brain and spinal cord [1,2,3]. It is characterized by the degeneration and death of motor neurons responsible for controlling voluntary muscle movements. The onset of ALS can vary from person to person, but common early symptoms include muscle weakness, spasms, and coordination difficulties. As the disease progresses, symptoms worsen, resulting in muscle weakness, atrophy, difficulties in speaking, swallowing, breathing, and eventually complete paralysis, and death [4,5]. In most cases, ALS does not affect intellectual abilities, and patients maintain cognitive abilities and continue to perceive their surroundings. Currently, there is no cure for ALS, and treatment focuses on managing symptoms, improving quality of life, and providing supportive care. The life expectancy of ALS patients varies; most people survive two to five years after the onset of symptoms [6,7]. However, some people may live longer, and a small percentage may experience a slower progression or even stable phases. Respiratory failure is the most common cause of death.

The exact cause of ALS is not well understood, but a combination of genetic and environmental factors is believed to play a critical role [8]. In about 5–10% of patients, ALS is inherited as a familial form (fALS), caused by mutations in specific genes [9], whereas about 90% of overall cases are sporadic forms (sALS) with no clear genetic association [10]. Other factors implicated in ALS pathogenesis include immune diseases [11,12,13,14], as well as viral infections, apoptosis, axonal degeneration, neuroinflammation, and glutamate-induced excitotoxicity [15]. Impaired glucose transport and metabolism in the brain, as well as defective glycogen metabolism and neuroglial metabolic interactions, also appear to be implicated in ALS pathogenesis [16]. Several mutations have been associated with fALS. (i) The expansion of a hexanucleotide repeat (HRE) in the open reading frame 72 (C9orf72) gene on chromosome 9 is responsible for a significant proportion of familial and sporadic ALS cases [17,18]. The exact mechanism by which the repeated expansion leads to neurodegeneration is still being investigated. (ii) Mutations in the superoxide dismutase 1 (SOD1) gene were the first genetic mutations linked to familial ALS [19]. They occur in approximately 12–23% of fALS cases and lead to complete inactivation of the enzyme, resulting in the accumulation of toxic protein aggregates and oxidative stress in motor neurons. (iii) Mutations in the TAR DNA-binding protein (TARDBP) gene, encoding a protein involved in RNA processing and regulation, have been found in approximately 4% of fALS cases [20]. (iv) Mutations in the fused in sarcoma (FUS) gene, an RNA-binding protein involved in several cellular processes, are responsible for about 4% of fALS cases [20]. (v) Mutations in the TDP-43 protein result in its nuclear depletion with mislocalization, misfolding, and the formation of cytoplasmic inclusion bodies in motor neurons. Indeed, phosphorylated and truncated forms of TDP-43 have been found in neuronal inclusions in over 97% of ALS patients, and are hallmarks of the disease [21]. (vi) Mutations in the ubiquilin 2 (UBQLN2) gene lead to the formation of abnormal protein aggregates that contribute to motor neuron dysfunction and are associated with a rare form of fALS known as ALS/ALS4.

However, these genetic mutations associated with fALS are not exclusive to ALS, as they have also been found in other neurodegenerative disorders, such as frontotemporal dementia (FTD). Therefore, various diagnostic tests are performed to rule out other conditions that can mimic ALS. A diagnosis of ALS is primarily based on clinical symptoms and a thorough neurological examination, and the procedures include electromyography (EMG), nerve conduction studies, muscle biopsies, spinal tap (lumbar puncture), and imaging studies like MRI or CT scans [22,23,24]. The diagnosis of fALS also makes use of genetic screening [25]. The lack of specific and early diagnostic biomarkers, especially for sALS, hinders pre-symptomatic diagnosis and delays treatment, which is critical to slowing disease progression in patients. Many research efforts are aimed at mitigating delays in diagnosis through biomarker development, considering that delayed detection also compromises patient enrollment in clinical trials, which requires the stratification of enrolled patients into homogeneous groups [26]. The identification of new clinical biomarkers for the early detection of ALS in a pre-symptomatic phase would be a major advance; some biomarkers could also be measured to monitor disease progression and be useful in promoting ALS treatment trials [27].

MicroRNAs are small, non-coding RNA molecules that regulate gene expression by binding to specific mRNA molecules and inhibiting their translation or promoting their degradation [28]. They play critical roles in various cellular processes, including development, differentiation, maintenance of neuronal function, and disease progression [29,30,31,32,33,34,35]. Most miRNAs found in the blood are tissue-specifically expressed, and circulating miRNA profiles can vary depending on physiological or pathological conditions; thus, they can serve as diagnostic and progression biomarkers for many types of diseases [36]. In the context of ALS, miRNAs are dysregulated in affected neural tissues, and their altered expression profiles may provide valuable insights into the pathogenesis of the disease [37,38]. Considering that about 70% of miRNAs are expressed in the brain [39], it is not surprising that they can be promising biomarkers for ALS [40]. An increasing number of studies are currently focused on their possible use as non-invasive diagnostic biomarkers for ALS in a pre-symptomatic stage [41,42].

In this work, we aimed to review and deepen the understanding of the use of miRNAs as useful clinical markers in ALS by analyzing in-depth original articles published over the last 16 years. We considered basic and translational research, including the potential clinical use of miRNAs as promising biomarkers for the early diagnosis of ALS and for monitoring disease progression. As a great number of studies on miRNAs and ALS are available in the literature; a systematic review approach was necessary to perform a deep and comprehensive investigation of the whole of the literature, especially when results were heterogeneous and/or controversial, as in this case. Indeed, while some miRNAs have consistently shown dysregulation in ALS across different studies, there is controversy regarding the specific miRNAs involved. Several studies have reported different miRNA expression patterns; on the whole, there is a lack of consensus on the specific miRNAs that play a significant role in ALS pathogenesis. This discrepancy may be due to differences in study design, sample size, tissue type, and patient heterogeneity. Another point of controversy is the tissue specificity of miRNA dysregulation in ALS. Some studies have focused on miRNA expression patterns in specific tissues, such as the spinal cord or skeletal muscle, while others have examined miRNAs in peripheral blood or cerebrospinal fluid (CSF). The variability in tissue-specific miRNA expression patterns adds complexity to the interpretation of results and the identification of reliable biomarkers for ALS. For this purpose, we adopted an innovative machine-learning-based approach by using the MySLR digital platform [43]. The latter is able to analyze a great number of papers through a text-mining method by employing the Latent Dirichlet Allocation (LDA) algorithm. This machine-learning-based classification approach allows us to locate studies, select and estimate quality contributions, analyze and combine results, and highlight clear conclusions, as already demonstrated in our previous work performed in the medical field [44]. In the present work, this strategy generated a systematic, comprehensive/integrated analysis of existing insights and knowledge gaps in current research, while also suggesting possible directions for future research.

## 2. Methods

The MySLR digital platform is a novel semi-automated tool supporting scientists in systematic literature review (SLR) performing [43]. It is available at https://myslr.unical.it (accessed on 26 January 2023, version 1.0, University if Calabria, Rende, Italy) upon registration. This tool represents an innovative machine-learning-based approach, as it is useful to simultaneously analyze a great number of scientific papers in the literature in order to reveal latent knowledge. While conventional algorithms are developed around numerical and structured data, the information generated in the scientific literature consists of generally unstructured documents (papers). Therefore, the LDA algorithm was adopted to extend machine-learning applications, thus extracting information from unstructured textual data consisting of scientific papers [45]. This platform, implementing the LDA algorithm, behaves almost like human intelligence; in fact, it is able to process a great number of data, analyze and understand text contents, extract the required information, and highlight hidden links among papers. By analyzing texts, a model is created that is able to autonomously generate a set of “topics” (or themes) within them. At the same time, the topic addressed by each of them is identified, as well as the presence of the topics identified above, within the various articles.

In the present work, we used this platform to obtain a complete SLR by analyzing scientific articles that, regardless of used medical protocols, somehow revealed potential links between miRNAs and ALS. Interestingly, studies carried out by adopting the same medical protocol are not contrasted by using this approach, as happens in conventional SLRs in the medical field. Consequently, we offered a comprehensive overview of the scientific research on the potential value of miRNAs as biomarkers in ALS. Our SLR was carried out according to the Preferred Reporting Items for Systematic Reviews and Meta-Analyses (PRISMA) guidelines [46].

Our methodological approach included three steps: paper location and selection, paper analysis, and results presentation [47]. A detailed description of each step is provided below.

### 2.1. Paper Location and Selection

We searched the PubMed, Scopus, and ISI Web of Knowledge online databases in order to identify relevant papers published in the field up to 26 January 2023. The terms associated with the keywords were (“microRNA” OR “miRNA” OR “miR”) AND (“Amyotrophic Lateral Sclerosis” OR “ALS”) AND “biomarker”. This search string was composed so that the results contained articles with at least one term from each set in the title, abstract, and keywords. Our search highlighted that the first paper in the investigated field was published in 2007.

### 2.2. Paper Analysis

At this stage, after removing duplicates, we examined the documents found (n = 308) in order to identify relationships and commonalities among them. Studies that met the following inclusion criteria were eligible:

- The study was performed on human cells and tissues, in animal models, or in patients with ALS.
- The study measured miRNA expression in blood, serum, plasma, urines, CSF, tissues, or cells.
- The study assessed the association between diagnosis and/or prognosis and miRNA expression.

Studies were excluded if:

- The study was a letter to editors, an editorial article, a personal opinion and commentary, a short communication, a meta-analysis, a review, a note, a book chapter, an erratum, or a retracted article.
- A study investigated other types of ALS biomarkers such as proteins, genes, or other types of non-coding RNAs with unknown functions, including long non-coding RNAs, circular RNAs, and small nucleolar RNAs.
- The study tested the prognostic role of target genes or other molecules instead of that of miRNAs.

We independently evaluated pertinent papers by examining titles, abstracts, and full texts matching the proper criteria. Upon completion of our selection, 89 papers were included in the final set of eligible documents for further topic extraction analysis. In order to highlight the main research topics in the context of miRNAs as potential clinical biomarkers in ALS, we used a text-mining method on the final set of 89 papers. This method employed LDA, a statistical algorithm that assigns each document a distribution along a certain number of topics. The model treats documents as topics of probability distribution and topics as word distributions.

In Natural Language Processing, a topic model represents a statistical model whose goal is to find the abstract “topics” (or themes) present in a set of documents. Topics are unknown a priori but are identified using the LDA algorithm, based on the frequency and number of occurrences of the words found in the screened texts. Therefore, we chose the best number of topics after examining the result of the LDA procedure, and identified 2 main topics related to the keywords detected by the LDA algorithm and present in the documents screened; furthermore, each text was correctly assigned its respective semantic topic. This procedure generally gives as outputs the following:

- k sets of pertinent keywords (each set denotes a topic).
- The document-term matrix, a matrix reporting how much each article is statistically related to a specific topic (known as topic proportion).

### 2.3. Results Presentation

The last step of our methodology is reported in the “Results” and “Discussion” sections. The purpose of this step is to properly explain and discuss the results of the LDA procedure through a comprehensive human-based review of the significant papers grouped into the topics.

## 3. Results

**Figure 1:**
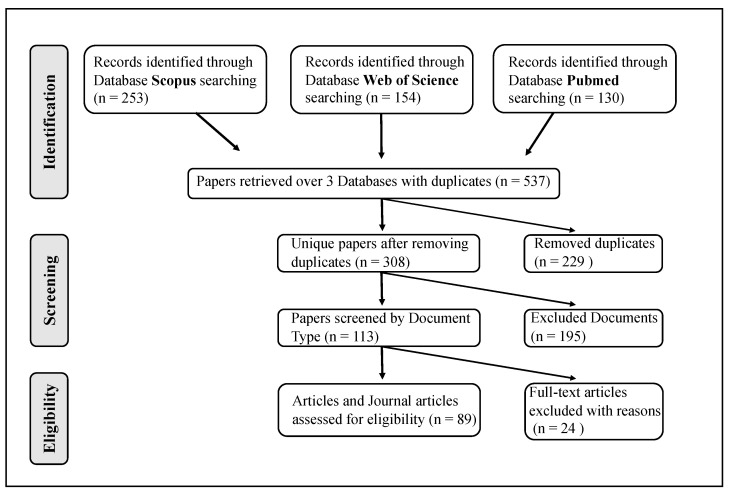
Flow diagram showing the algorithm of selection of eligible studies included in the SLR. The search process was carried out until 26 January 2023.

A total number of 537 papers were retrieved from the preliminary literature search, and 308 after the removal of duplicates. Of these, 195 papers were removed as they consisted of irrelevant studies such as editorials, reviews, meta-analyses, short surveys, book chapters, and other types of irrelevant publications. Full-text reading and analysis led to the removal of 24 further papers for reasons including inability to access the full text, papers not written in English, or not meeting the inclusion criteria mentioned above. Finally, 89 studies were considered to be eligible. The flowchart reported in Figure 1 depicts the selection algorithm of the final studies included in this systematic review.

**Figure 2:**
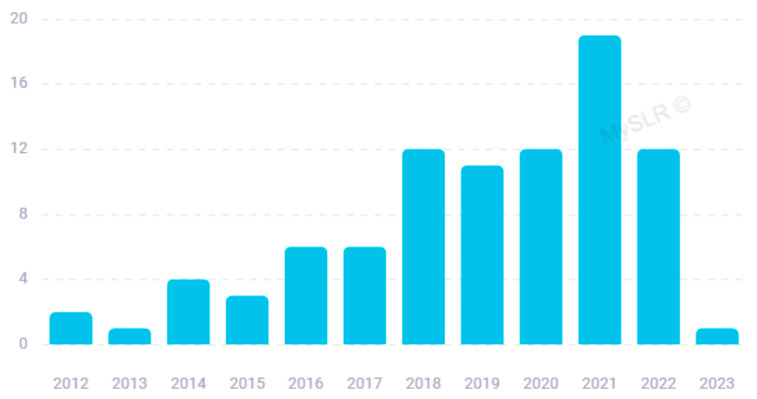
Published articles by year.

Scientific interest in this issue has generated many original articles over time, starting from 2012 (Figure 2); if we do not consider 2023, which is still ongoing, most of the articles have been published in the last 5 years. Based on the indication provided by Blei et al. [45], we chose the k value (number of topics to be extracted) of two, which ensured a reasonable value of topic coherence (−1.09) [48] in accordance with an easy interpretation of the results. On account of the LDA procedure, we identified significant keywords associated with each topic.

**Figure 3:**
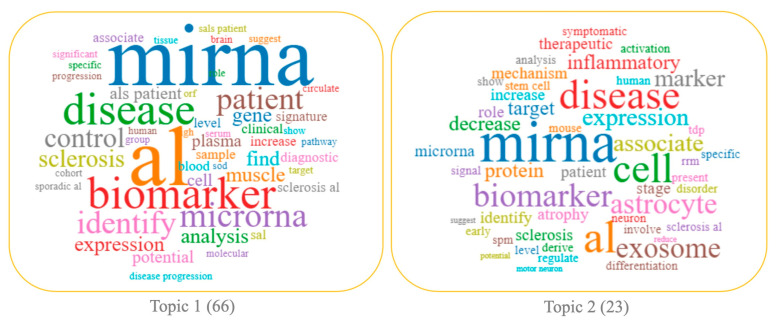
Word clouds highlight the importance of the keywords for each topic.

In Figure 3, a graphical representation of the most relevant keywords found for each topic has been reported as a “word cloud”.

**Figure 4:**
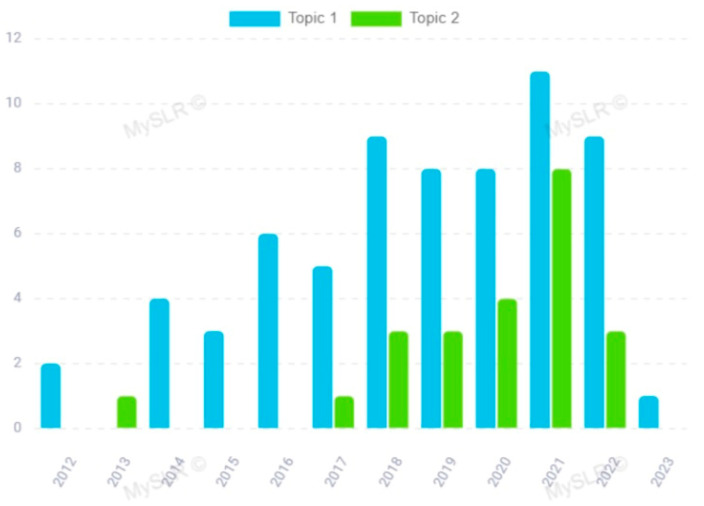
Topic papers over time.

Although the issue under investigation has been scientifically attractive in recent years, the trend of the topic papers over time is quite different (Figure 4). In detail, topic 1 (Figure 4, blue) aroused progressively growing scientific interest over time, albeit in a fluctuating way; in fact, in 2013 it was not attractive at all. The scientific interest in topic 2 (Figure 4, green) was evident in 2013; it had a recovery in 2017 until reaching its peak in 2021, and then perhaps interests were lost.

The two topics identified using the LDA algorithm have been discussed below. In particular, all the papers, selected by the machinelearning procedure and clustered in each topic, have been manually analyzed by all of the research team so that poorly designed studies have been excluded from the analysis. Thus, we performed a human-based analysis of a subset of pertinent papers in order to obtain a significant description of each topic. The numbering of the two topics was established by the software. Following a logical order, we decided to discuss topic 2 first, as it deals with aspects of basic research such as the involvement of miRNAs in the pathogenesis, development, and progression of the disease, whereas topic 1 concerns the potential of miRNAs in translational and clinical research.

### 3.1. Topic 2—miRNA in ALS Pathogenesis

**Figure 5:**
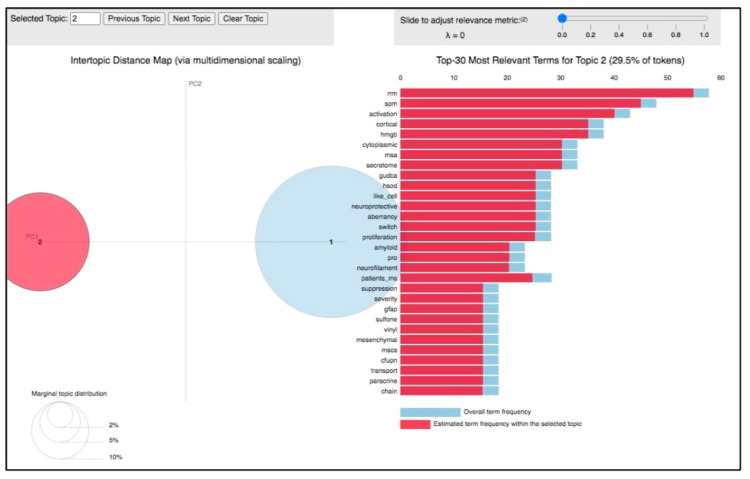
Inter-topic distance map related to topic 2. Circle 1 indicates topic 1 and circle 2 is topic 2.

By analyzing the 23 papers clustered in this topic, and by taking a quick look at the top 30 most relevant terms and their frequency within the papers grouped around the selected topic (Figure 5), it was evident that the essence of the topic was the involvement of miRNAs in ALS progression and development. Indeed, most of the papers clustered in the topic, and summarized in Table 1, share results obtained from in vitro and in vivo experiments, such as in animal ALS models. These works explore the function of dysregulated miRNAs, their target genes, and the main regulatory pathways implicated in ALS (Table 1).

The dysregulation of miRNAs and their effects on target genes contribute to the complex molecular mechanisms underlying disease pathogenesis through various mechanisms, including neuronal cell loss, neuroinflammation, glial cell dysfunction, protein aggregation, and mitochondrial dysfunction, thus leading to the progressive loss of motor neurons that is characteristic of ALS.

Among the microRNAs identified by our analysis, miRNA-124 is one of the most extensively studied in ALS. It is primarily expressed in the nervous system and plays a critical role in neuronal differentiation and maturation. ALS patients often exhibit an upregulation of miRNA-124 and higher expression levels have been associated with slower disease progression and better survival outcomes [49]. Also, the upregulation of the miRNA-338-3p family in ALS patients may lead to the downregulation of their target genes, which are involved in neuronal cell survival and maintenance [50]. The deregulation of miRNAs can impact the inflammatory response by modulating the expression of genes involved in immune regulation and neuroinflammation. Elevated miRNA-146a and miRNA-155 levels, particularly in the spinal cords of ALS patients, for instance, can target genes involved in the regulation of immune responses and contribute to the neuroinflammatory processes observed in the disease [51,52,53,54]. The dysregulation of miRNAs can affect glial cells such as astrocytes and microglia, which play critical roles in supporting neuronal health and contribute to ALS pathogenesis. Altered miRNA expression in glial cells may influence their inflammatory response and secretion of neurotoxic factors, as well as impairing the clearance of toxic protein aggregates, all of which may contribute to motor neuron degeneration. For example, miRNA-9 is involved in neuronal development, synaptic plasticity, and neuroprotection, and it has been shown to target TDP-43 and modulate its expression levels. The dysregulation of miRNA-9 in ALS patients may interfere with the normal clearance of TDP-43, leading to its abnormal accumulation and subsequent neurotoxicity [55]. Also, altered miRNA expression may contribute to mitochondrial dysfunction observed in ALS and further exacerbate neuronal cell death [56]; in this regard, the dysregulation of miRNA-23a is able to impair mitochondrial function by regulating the expression of genes involved in mitochondrial dynamics, energy production, and oxidative stress response.

Therefore, identifying miRNAs dysregulated in ALS and understanding the miRNA-mediated mechanisms can provide insights into the underlying pathogenesis of ALS and potentially identify novel therapeutic targets for intervention.

### 3.2. Topic 1—miRNA in the Clinical and Translational Research of ALS

**Figure 6:**
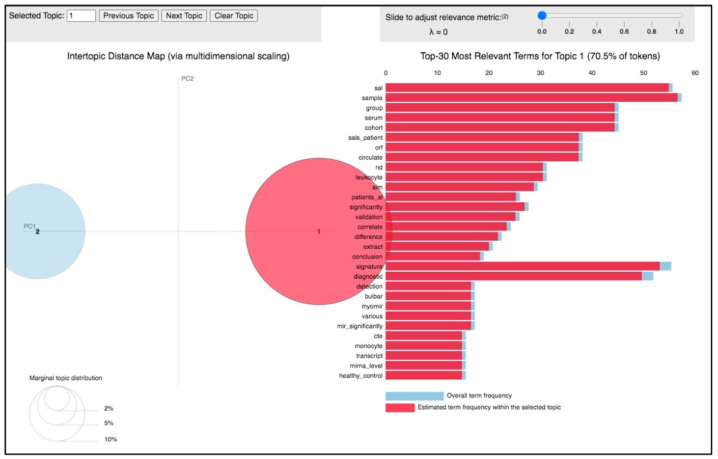
Inter-topic distance map related to topic 1. Circle 1 indicates topic 1 and circle 2 is topic 2.

The analysis of the 66 papers clustered in topic 1 revealed its distinctive feature was the potential use of miRNAs in the clinical setting as promising diagnostic biomarkers for ALS. This was also supported by the particular terms present in the papers clustered on this topic, including “patient”, “biomarker”, “analysis”, “control”, “diagnostic”, “signature”, “clinical”, “muscle”, “plasma”, “serum” (Figure 3), and by the top 30 most relevant terms found in the papers of this topic (Figure 6). This research area is in rapid development; indeed, topic 1 is based on a growing body of studies whose scientific interest has increased in the considered period, especially in 2018 and 2021 (Figure 4, blue and Table 2).

The studies clustered in this topic evaluated the expression profiles of many human miRNAs, comparing ALS patients, healthy subjects, and sometimes patients with other neurodegenerative diseases. In these studies, ALS-associated miRNA profiles were evaluated mostly by next-generation sequencing (NGS), microarray analysis, and reverse transcription quantitative PCR (qRT-PCR) performed in different patient populations. The search for miRNAs as ALS biomarkers was mostly performed in biofluids such as CSF [60,61] and blood (plasma or serum) [58,62,63,64,65], also evaluating peripheral blood leukocytes and blood extracellular vesicles (EVs) that are secreted by neural cells and cross the blood–brain barrier, reaching the bloodstream [66,67,68,69]. Some miRNAs were also extracted from muscle biopsies of ALS human donors [70] or analyzed on spinal cord or motor cortex tissues [71].

Several miRNAs have been investigated as potential biomarkers for the diagnosis and prognosis of ALS. Our analysis selected those deemed promising in clinical and translational research. We schematized the results in Table 2.

MiRNA-124 was found in the brain and is involved in neuronal differentiation and function. Elevated levels of miRNA-124a have been found in the CSF and serum of ALS patients, and have been associated with disease severity and shorter survival, suggesting that it could be a potential prognostic marker for disease progression [72,73]. The dysregulation of miRNA-155 may reflect the immune system response to ALS pathology and could be a potential biomarker for disease monitoring and prognosis. Indeed, its increased expression has been detected in the peripheral blood mononuclear cells (PBMCs) of ALS patients and has been associated with disease progression and survival [74,75]. MiRNA-9 plays a critical role in neuronal development and synaptic plasticity. A reduced expression of miRNA-9 has been observed in the motor cortex of ALS patients. Since its downregulation may contribute to neurodegeneration and impaired neuronal function in ALS, monitoring miRNA-9 levels could provide insights into disease progression and help to identify molecular targets for therapeutic interventions [68,73]. MiRNA-206 is muscle-specific and is involved in muscle development, regeneration, and maintenance. An increased expression of miR-206 has been observed in the skeletal muscle and serum of ALS patients and has been associated with disease severity, muscle atrophy, and a faster progression of ALS symptoms, thus suggesting its potential as a diagnostic and prognostic marker [58,76,77,78,79,80,81]. Magen et al. recently highlighted that elevated plasma levels of miRNA-181 predicted an increased risk of death in an independent discovery patient cohort and a replication patient cohort, thus highlighting that miRNA-181 could represent a validated biomarker for ALS prognosis [82]. According to these authors, miRNA-181’s performance was similar to that of neurofilament light (NFL); moreover, superior prognostic ability was achieved by the combined measurements of both these biomarkers, thus promising to improve the accuracy of patient stratification for future clinical trials. Furthermore, these authors demonstrated that miRNA-181 was largely expressed in neurons in the mouse motor cortex and spinal cord, suggesting that it could be a biomarker of axonal damage as NFL [82]. The latter is a biomarker present in the CSF and consequently in the blood; it is negatively associated with patient survival and is considered a clinically validated prognostic biomarker for ALS [83]. Lastly, the expression of miRNA-338-3p was found to be upregulated in several studies, and in different sample types from sALS patients, such as blood leukocytes, CSF, serum, and the spinal cord, thus supporting the hypothesis that it could represent a very interesting candidate diagnostic biomarker [71,84,85]. Other studies have shown that its expression is reduced in the leukocytes of ALS patients and that its decreased levels are correlated with shorter survival and a more aggressive disease course [73,86]. These contrasting results could depend on the specific study population, sample type, and analytical techniques used. Thus, although this miRNA has shown potential as a biomarker in ALS, further research is needed to establish standardized protocols for its measurement in order to validate its utility.

**Table 2:** List of the most relevant and representative miRNAs (miR) highlighted in papers clustered in topic 1. ↑ indicates upregulated miR; ↓ indicates downregulated miR.

Dysregulated miRNAs	Biological Sample	Methods for miRNA Profiling	Clinical Value	References
↑ miR-124	Exosomes of CSF; Blood leukocytes	RT-qPCR	Increased expression has been associated with disease severity and shorter survival	[72,73]
↑ miR-155	Peripheral Blood; Mononuclear Cells (PBMCs); Spinal cord	Microarray technology; RT-qPCR	Elevated levels have been associated with disease progression and survival	[74,75]
↓ miR-9	Blood leukocytes; EVs	RT-qPCR; Next-Generation Sequencing	Reduced expression has been associated with disease severity and shorter survival	[68,73]
↑ miR-206	Serum	RT-qPCR	Higher levels associated with disease progression and muscle atrophy	[58,76,77,78,79,80,81]
↑ miR-181	Plasma; CSF	Next-Generation Sequencing	High levels predict a greater than two-fold risk of death in independent discovery and replication cohorts	[60,82]
↑ miR-338-3p	Blood leukocytes; CSF; serum and spinal cord	Next-Generation Sequencing; Meta-analysis using rank aggregation (RRA) method, followed by RT-qPCR validation.	Elevated levels have been associated with disease progression and survival	[71,84,85]
↓ miR-338-3p	Blood leukocytes	Microarray technology	Decreased levels correlated with shorter survival and a more aggressive disease course	[73,86]

As mentioned above, this specific research field is still evolving; therefore, further validation and larger-scale studies are necessary to identify robust miRNA biomarkers useful for ALS diagnosis, prognosis, and monitoring of disease progression to establish their effective clinical utility.

## 4. Discussion

In the context of ALS, miRNAs have been extensively studied to understand their involvement in the disease, being implicated in various aspects of ALS pathogenesis, including neuroinflammation, oxidative stress, excitotoxicity, protein aggregation, mitochondrial dysfunction, and defective RNA metabolism. Dysregulated miRNAs can contribute to the impairment of these cellular processes, leading to motor neuron degeneration and disease progression. ALS pathogenesis is not yet sufficiently understood, one of the reasons that hinder the understanding of the disease is its considerable heterogeneity from a genotypic and phenotypic point of view. It is known that the dysregulation of TDP-43 and SOD1, both implicated in the biogenesis of miRNAs, could play a crucial role. Their roles have been extensively studied in ALS pathogenesis. TDP-43 misfolding and pathology in motor neurons have been found in most ALS cases [21]. Although SOD1 impairs RNA stability and function, animal models and patients with SOD1 mutation do not show TDP-43 alterations [21]. However, previous studies have detected alterations in several miRNAs that are common in SOD1 transgenic mice and sALS patients. This implies that functional alterations of common miRNAs, regardless of the responsible proteins, contribute to ALS pathogenesis and progression, interfering in critical cellular pathways. Nonetheless, more detailed validation of ALS models other than SOD1 transgenic mice is needed to test this hypothesis.

Moreover, the role of miRNAs in the clinical and translational research of ALS has been a subject of significant interest. Due to their stability, presence in various body fluids, and specific expression patterns, microRNAs are believed to be potential clinical markers for ALS. Indeed, dysregulated miRNA expression profiles have been identified in various biological samples, including blood, urine, CSF, and skeletal muscle from ALS patients. In this regard, CSF is the main reservoir of neural cell by-products from degenerated cell metabolism, thus representing a major source of potential biomarkers in ALS. On the other hand, CSF is not as easily obtainable in blood. Recently, some authors highlighted that the use of CSF as a source of miRNAs that are potentially useful as diagnostic biomarkers is controversial. More specifically, Joilin et al. [61] identified by RNA-sequence analysis numerous significantly dysregulated miRNAs in the CSF from ASL patients compared to healthy controls, including let-7c-5p, miRNA-9-3p, miRNA-196a-5p, miRNA-211-5p, miRNA-451a, and miRNA-6797-5p. Furthermore, miRNA-494-3p, miRNA-885-5p, and miRNA-6501-5p were found to be dysregulated in both slow- and fast-progressing ALS patients with respect to healthy controls. However, the dysregulation of all these miRNAs was not validated by qRT-PCR assays performed on individual samples, probably due to the low amounts present in the samples, so their utility as ALS biomarkers was not confirmed. Notably, these authors supposed that blood represented a more reliable sample than CSF, as in CSF they did not detect the upregulation of miRNA-206, although it had been detected in the blood in previous studies performed by the same authors; in addition, they could not confirm the dysregulation of other miRNAs previously proven to be dysregulated in ALS serum [81]. Other studies found no correlation between dysregulated miRNAs detected in serum and with those identified in CSF. In this regard, Freischmidt et al. [87] found a poor correlation between CSF and serum levels of some dysregulated miRNAs in sALS patients. In detail, significant decreases in the expression levels of miRNA-132-5p, miRNA-132-3p, and miRNA-143-3p, as well as increases in those of miRNA-143-5p and miRNA-574-5p were measured in sALS CSF samples when compared to healthy controls, also let-7b levels were decreased but without statistical significance. Significant decreases in the expression levels of miRNA-132-5p, miRNA-132-3p, miRNA-143-5p, miRNA-143-3p, and let-7b were found in the serum samples of the same patients. On this basis, the authors hypothesized the existence of an independent regulation of these TDP-43-binding miRNAs in the bloodstream and CSF [87]. Overall, existing studies on CSF-isolated miRNAs as diagnostic and prognostic biomarkers of ALS were small in scale and not reproduced; moreover, they were not aggregated in order to evaluate expression profiles of miRNAs from EVs. Furthermore, several extraction and sequencing methods were used for the detection of miRNAs from CSF, but the best ones have not yet been determined [88]. Very recently, saliva has gained increasing interest as a source of EVs, due to its non-invasive and simple collection access. Salivary EVs seem to have higher purity than those found in plasma, and can be isolated by using membrane affinity-based methods [89]. This method could offer significant opportunities and strategies to isolate and validate miRNA signatures for ALS detection and prognoses worthy of further investigation for clinical use. More recently, blood EVs have been studied for early non-invasive ALS diagnosis, as they represent a brain “liquid biopsy” for evaluating neurodegenerative diseases; indeed, specific molecules indicating the cells of origin are present on their surface, as well as molecular signatures reflecting cellular states and metabolism in pathological conditions. On this basis, changes in the expression/concentration of disease-associated molecules such as miRNAs contained in EVs can be studied to find a specific signature of dysregulated miRNAs in ALS, which could be useful for early diagnosis and monitoring the disease [67,68]. The analysis of EV-miRNAs appears to be convenient, as EVs are a stable source of miRNAs in biofluids, thus providing consistent miRNA expression levels as well as resistance to RNase degradation and the possible effects of factors affecting miRNA expression. Furthermore, miRNAs inside EVs are free from irrelevant background molecules such as serum proteins, which can improve sample quality for subsequent analyses [90]. EVs can be obtained from patients’ blood and CSF by using a slightly invasive approach, and then isolated [91]. The use of extraction polymer-based techniques to isolate EVs was believed to be more reproducible [66]. EVs express cell-specific proteins on their surface; thus, subpopulations of specific origins could be enriched by using immunological methods, such as the transmembrane L1 cell adhesion molecule (L1CAM) antibody affinity procedure, which was employed to obtain neural-enriched extracellular vesicles (NEE), as reported in several recent studies [66,69]. Sproviero et al. used filtration, differential ultracentrifugation, and Western blot analysis to isolate small and large EVs (SEVs and LEVs, respectively) circulating in the blood of patients with different neurodegenerative diseases, including ALS, fronto-temporal dementia, Alzheimer’s disease, and Parkinson’s disease, thus detecting the presence of a huge number of dysregulated miRNAs [92]. Based on the studies described, it is clear that the protocols adopted to isolate EVs have been different. The standardization of EVs’ isolation is a critical point, and currently it is required to obtain reproducible analyses and achieve consensus results. Indeed, the lack of reproducibility between studies has hindered the introduction of miRNAs extracted from EVs in the clinical setting. To date, EVs’ isolation performed by using L1CAM has been controversial; recent findings have highlighted that L1CAM immune-capture can offer better reproducibility than other methods [93]. Conversely, other authors have demonstrated that L1CAM expression is not restricted to neurons, the isoforms found in the CSF and plasma are different, and that that found in plasma might not be associated with EVs; thus, L1CAM-based methods should not be employed for the isolation of neuron-derived EVs [94].

Overall, the main limitations of all the studies conducted in order to find validated miRNAs useful as ALS biomarkers are due to inconsistencies between them; this has caused a lack of uniformity and consensus on these biomarkers, as well as on the direction of their regulation. These discrepancies may result from different sampling, processing, and assay protocols used in different laboratories, or from the heterogeneous nature of ALS. Additional issues are the small sizes of the cohorts studied, the lack of investigation into the influence of lifestyle factors interfering with microRNA levels in the studied samples, and possible differences in ALS-related miRNA profiles due to the tissues/samples employed for the analysis, in agreement with what was observed by some authors [41]. From a reproducibility perspective, validation studies using independent datasets are essential for determining the utility of candidate miRNAs as robust biomarkers for ALS. Furthermore, few longitudinal studies have been performed in this regard.

Extensive studies over the last 12 years have clearly suggested that several miRNAs are differentially expressed in ALS patients compared to healthy individuals or patients with other neurodegenerative disorders, that they could provide insights into the complex molecular mechanisms underlying ALS, and that they have the potential to serve as valuable biomarkers for diagnosis, prognosis, and targeted therapies. As previously mentioned, miRNA expression profiles can vary between studies due to differences in patient cohorts, methodologies, and experimental conditions; therefore, these studies are often controversial. Some questions are being answered, while many new ones need to be answered. Our work, using a machine-learning approach, aimed to deepen the understanding of the existing scientific literature. Based on the LDA algorithm, we found two main topics that the literature focused on, and that are also areas for future research. Our analysis leads us to conclude that several miRNAs are associated with ALS pathogenesis. For example, miRNA-124, miRNA-155, and miRNA-206 have been consistently reported to be upregulated in ALS patients, while miRNA-9 has been found to be downregulated. These miRNAs can serve as non-invasive biomarkers for the early and accurate diagnosis of ALS. MicroRNAs may also help to monitor disease progression and prognosis. Longitudinal studies have shown that certain miRNA expression profiles correlate with disease severity, progression rate, and survival. For example, miRNA-124 has been associated with slower progression and better survival, whereas miRNA-338 has been associated with fast disease progression and poor prognosis. Furthermore, monitoring the expression levels of these miRNAs over time can provide valuable prognostic information to help to predict clinical progression and stratify patients into subgroups with different disease characteristics for clinical trials or personalized medicine approaches.

Therefore, the dysregulation of miRNAs in ALS opens opportunities for the development of novel miRNA-based therapeutic strategies. Translational research efforts, as previously shown, are moving in this direction and are focused on optimizing delivery methods to target tissues and assessing their safety and efficacy in clinical settings. Modulating the expression of specific miRNAs holds the potential for restoring normal gene expression patterns and slowing down disease progression or even reversing neurodegeneration. Several strategies have been explored, such as miRNA replacement therapy, which consists of using miRNA mimics to restore the levels of downregulated miRNAs to normal expression patterns, or using anti-miRNA oligonucleotides to inhibit the activity of upregulated miRNAs. These approaches have shown promising results in preclinical studies using animal models, highlighting the therapeutic potential of miRNAs as targets for ALS treatment.

## 5. Conclusions

In conclusion, miRNAs play an important role in clinical and translational ALS research as potential biomarkers for diagnosis and prognosis and are increasingly explored as therapeutic targets. Their altered expression profiles in ALS patients provide a non-invasive and readily accessible means of diagnosing the disease. Monitoring specific miRNAs over time can help to predict disease progression and guide treatment decisions. However, there are still controversies and challenges regarding their role in disease pathogenesis. Research in this area is still ongoing to better understand the specific miRNAs, their targets, and their regulated pathways, in order to validate their functional significance in disease development and progression. It remains challenging to determine whether the observed dysregulation of miRNAs is a cause or a consequence of the disease. Further research is needed to evaluate whether specific miRNA signatures can be specifically used for early and non-invasive diagnosis of ALS and for predicting progression rate. In particular, the standardization of protocols, the use of larger sample sizes, and more comparisons with blood samples from ALS-like diseases are needed to evaluate the specificity and sensitivity of candidate biomarkers. On this basis, miRNA analysis performed by using reproducible techniques could make them promising candidates for further research aimed at clinical application. Therefore, research efforts will need to be focused on developing standardized protocols for their measurement in order to validate the clinical utility of miRNAs and translate these findings into routine clinical practices for the benefit of ALS patients.

## Figures and Tables

**Table 1:** List of the most relevant and representative dysregulated miRNAs (miR) highlighted in the papers clustered in topic 2. ↑ indicates upregulated miR; ↓ indicates downregulated miR.

Dysregulated miRNAs	Target Genes	Main Regulatory Effects	References
↑ miR-124	C/EBPα (CCAAT/enhancer-binding protein alpha), STAT3 (Signal transducer and activator of transcription 3)	Regulates neuronal differentiation and maturation; dysregulation may contribute to neuroinflammation and neurodegeneration in ALS	[49]
↑ miR-338-3p	PYGB (Glycogen Phosphorylase Brain)	Dysregulation leads to decreased glycogenolysis and subsequent aberrant accumulation of glycogen that causes glucotoxicity and impairs neuronal functions, leading to neurodegeneration	[50]
↑ miR-146a	IRAK1 (Interleukin-1 receptor-associated kinase 1), TRAF6 (TNF receptor-associated factor 6)	Regulates immune responses and inflammation; dysregulation may contribute to neuroinflammatory processes in ALS	[51,52,53]
↑ miR-155	TAB2 (TGF-beta-activated kinase 1/MAP3K7-binding protein 2), SOCS1 (Suppressor of cytokine signaling 1)	Involved in immune regulation and neuroinflammation; dysregulation may contribute to glial cell dysfunction and disease progression in ALS	[54]
↓ miR-9	TDP-43 (Transactive response DNA-binding protein 43)	Dysregulation may disrupt the normal clearance of TDP-43, leading to its accumulation and subsequent neuroinflammation and neurotoxicity	[55]
↑ miR-23a	PGC-1α (Skeletal muscle peroxisome proliferator-activated receptor γ coactivator-1α)	Regulates signaling networks involved in mitochondrial biogenesis and function	[56]
↑ miR-206	HDAC4 (Histone deacetylase 4), Cx43 (Connexin 43)	Regulates muscle regeneration and repair, and its dysregulation may contribute to muscle atrophy and impaired motor function in ALS	[57]
↑ miR-143	FUS (Fused in sarcoma), SMAD3 (Mothers against decapentaplegic homolog 3)	Involved in RNA metabolism and cellular stress response; dysregulation may contribute to the accumulation of abnormal protein aggregates and neuronal cell death in ALS	[58]
↑ miR-26a	SLC1A1 (Glutamate transporter)	Regulates the glutamate receptor signaling pathway. Dysregulation leads to high levels of glutamate in the CNS, causing inflammation and neurodegeneration	[59]
